# Colony Stimulating Factor 1 Receptor in Acute Myeloid Leukemia

**DOI:** 10.3389/fonc.2021.654817

**Published:** 2021-03-25

**Authors:** Kristine Yttersian Sletta, Oriol Castells, Bjørn Tore Gjertsen

**Affiliations:** ^1^ CCBIO, Centre for Cancer Biomarkers, Department of Clinical Science, Precision Oncology Research Group, University of Bergen, Bergen, Norway; ^2^ Department of Medicine, Hematology Section, Haukeland University Hospital, Bergen, Norway

**Keywords:** colony stimulating factor 1 receptor, tumor-stroma, signal transduction, biomarkers, acute myeloid leukemia, targeted therapy, therapy development

## Abstract

Acute myeloid leukemia (AML) is an aggressive heterogeneous blood cancer derived from hematopoietic stem cells. Tumor-stromal interactions in AML are of importance for disease development and therapy resistance, and bone marrow stroma seem like an attractive therapeutic target. Of particular interest is colony stimulating factor 1 receptor (CSF1R, M-CSFR, c-FMS, CD115) and its role in regulating plasticity of tumor-associated macrophages. We discuss first the potential of CSF1R-targeted therapy as an attractive concept with regards to the tumor microenvironment in the bone marrow niche. A second therapy approach, supported by preclinical research, also suggests that CSF1R-targeted therapy may increase the beneficial effect of conventional and novel therapeutics. Experimental evidence positioning inhibitors of CSF1R as treatment should, together with data from preclinical and early phase clinical trials, facilitate translation and clinical development of CSF1R-targeted therapy for AML.

## Introduction

Acute myeloid leukemia (AML) is the most common aggressive blood cancer in adults with a median age at diagnosis of 71 years and with an overall incidence of approximately 4/100.000. In patients older than 70 the incidence is 17/100 000 (1). The 5-year survival rate for AML was less than 20% (2) before a wave of FDA-approved novel agents (introduced from 2017) were incorporated into standard treatment regimens and entered late-stage development in therapy combinations (3, 4). Even with these recent improvements, there have been few therapy developments that address tumor-host interactions. It is foreseen that AML will continue to represent a therapeutic challenge requiring novel treatment modalities (5).

AML is characterized by disruptive hematopoiesis through block in myeloid differentiation and enhanced proliferation leading to accumulation of non-differentiated myeloid cells/myeloblasts in bone marrow and peripheral blood (6). AML is diagnosed when myeloblast count comprise at least 20% of the bone marrow (7). Normal blood production is interrupted, and a typical AML patient may present low numbers of functionally intact granulocytes, platelets, and erythrocytes. Symptoms often include fatigue, shortness of breath, easy bruising, and frequent infections.

AML is a heterogeneous disease comprising both recurrent and rare chromosomal translocations and mutations. Next generation sequencing (NGS) analyses of leukemic samples have contributed to reveal the genetic landscape of AML, showing an enormous mutational diversity and identifying over 30 recurrent mutations (7–10). In some subsets of AML, molecular diagnostics can suggest the therapies most likely to give a favorable outcome such as expression of cell surface marker CD33 or mutations in specific genes such as FLT3/IDH1/IDH2 (8, 11, 12). Among the most frequent mutations in AML are those affecting nucleophosmin 1 (NPM1) which is possible to target experimentally, as recently demonstrated (13–16). Similarly, mutations in TP53 are associated with chemo-resistance and therapies aimed at restoring P53 function are in development (17).

It has been postulated that the process of leukemogenesis is initiated by relatively few steps, and a “two hit” model of AML development has been proposed, in which class 1 and class 2 mutations can suffice for AML initiation (18). This theory has mostly been supported by the analyses of large AML sample cohorts (10, 19). Observations indicate that AML is a disease that starts with a single clone that acquires novel mutations over time, thereby further contributing to tumor heterogeneity and early relapse (20–22).

The acquisition of somatic mutations is a relatively common event in most cell types and increases with age (23, 24). Certain mutations occur in hematopoietic stem cells (HSC) and gain a competitive advantage, resulting in “clonal hematopoiesis” that could lead to expansion of a clonal population of blood cells (25). Clonal hematopoiesis is predisposing individuals to hematological disease (26, 27) but studies have discovered that clonal hematopoiesis-harboring mutations in AML-associated genes like DNMT3A and TET2 are ubiquitous in the elderly population between 50 and 70 (28). Although prevalence of clonal hematopoietic mutations is very common, progression to hematological malignancy is extremely rare. A recent investigation found no significant association between clonal hematopoiesis and long-term risk of developing AML between cases and controls (29). Future research should rely on methods to distinguish between high-risk and low-risk clonal mutations for development of aggressive disease. This imperative should gain interest in sequencing-based non-invasive screening and in future tailored therapy guided by cytogenetics and mutational profile.

The AML therapies implemented from 2017 filled a nearly 40-year paucity in drug development (12). These new agents include lipid formulated chemotherapy, antibodies directed against AML cells, Bcl-2 family inhibitors, metabolic enzyme inhibitors of IDH1/2, and tyrosine kinase inhibitors. Although developing targeted therapy presents challenges, the accumulated knowledge about AML will continue to translate into novel treatment approaches that improve patient outcomes (30). In fact, treatment based on molecular diagnostics should increase overall survival of AML above the glass ceiling of 50% long-term survival currently observed in younger and fit patients (7, 31). The recent therapeutic landscape of AML does not include stroma-targeting therapy, although intensive induction chemotherapy and consolidating allogeneic stem cell transplantation eradicate most of the host stromal environment. At the same time, it appears clear that broad acting kinase inhibitors like midostaurine and gilteritinib (predominantly inhibitor of FLT3, AXL) may affect stromal function (32).

Colony Stimulating Factor 1 receptor (CSF1R) is a particularly interesting target since its expression and signaling is prominent in the supportive stromal compartment. The use of enzymatic inhibitors and blocking antibodies of CSF1R represents two novel approaches that addresses tumor-stroma interactions in an attractive way.

## CSF1R Biology

**Figure 1:**
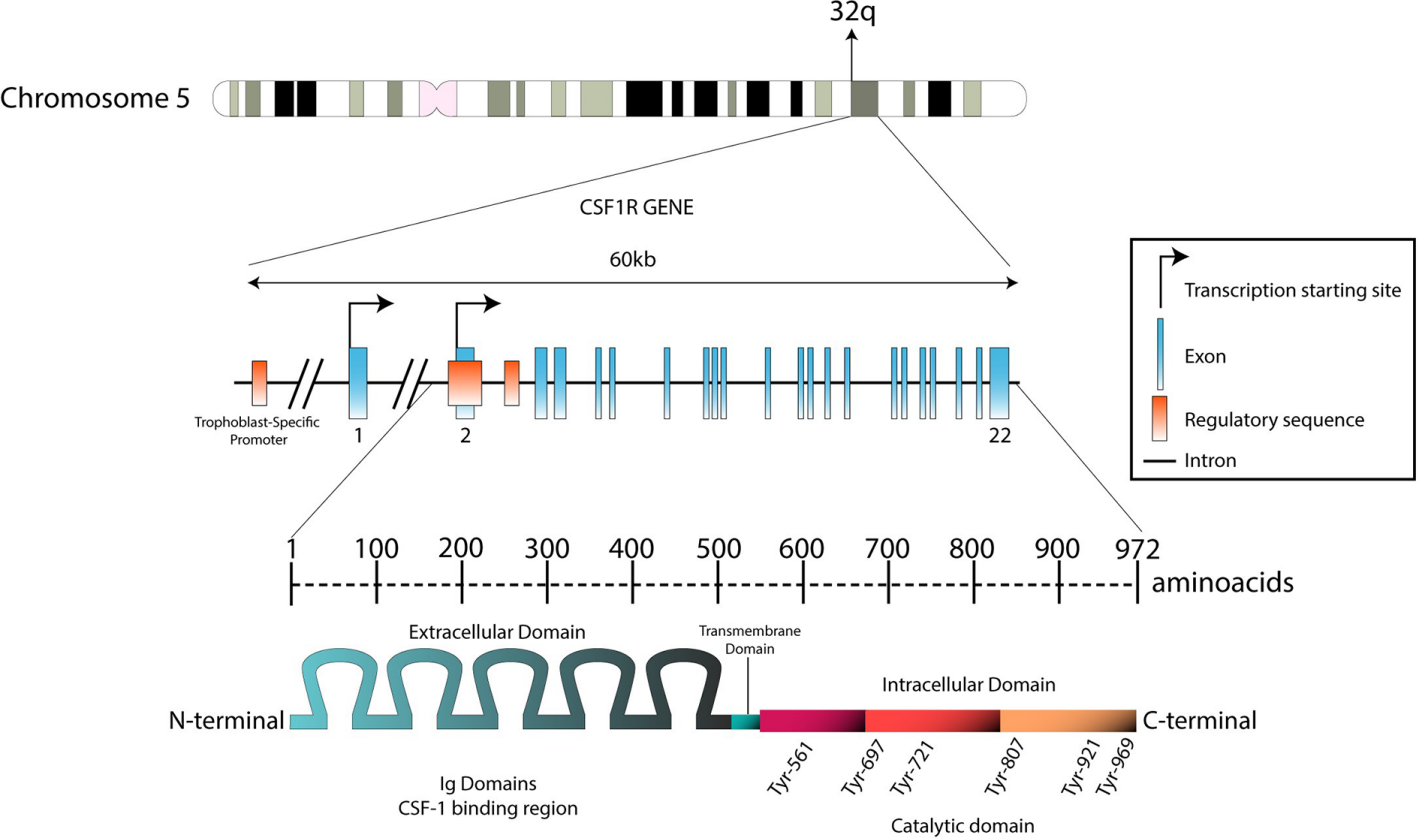
Genomic structure of the CSF1R locus in human and protein structure. The CSF1R locus is located at the distal end of the q arm in chromosome 5 (5q32). With 60 kb length, CSF1R gene is composed by 22 exons. Transcription between the first exon and exon 22 is exclusively regulated by a trophoblast-specific promoter. In other tissues (such as macrophages), transcription takes place only between exon 2 and exon 22. The transcript produced is predicted to be 3.9 kb long. This product will translate in a 972-aminoacid protein with a molecular weight of 108 kDa. The N-terminal extracellular domain is composed by five immunoglobulin domains (512 aa), which contain the ligand binding region. The hydrophobic transmembrane domain is 25 amino acids long. The intracellular domain contains the tyrosine residues that will be phosphorylated upon receptor stimulation (435 aa).

CSF1R (M-CSFR, c-FMS, CD115, c-fms proto-oncogene, McDonough feline sarcoma oncogene) is a cell surface glycoprotein encoded by the CSF1R gene located on the distal end of the long arm chromosome 5 (5q32) (**Figure 1**) (33). CSF1R is a class III receptor tyrosine kinase and member of the platelet-derived growth factor (PDGF) receptor family along with FLT3, c-KIT, and PDGF-α and -β receptors (34). In comparison, CSF2R (GM-CSFR, CD116) belongs to class 1 hematopoietic receptor systems and CSF3R (G-CSFR, CD-114) is related to the cytokine (hematopoietin) receptor family (35). CSF1R is expressed primarily on mononuclear phagocytes, namely monocytes, macrophages, and dendritic cells where its activation is crucial for their growth and differentiation during immune responses. CSF1R is involved in promoting the physiological properties of monocytes and macrophages which entail cytotoxicity, phagocytosis, and chemotaxis through the release of cytokines and chemokines. CSF1R is also found on a diversity of cells of the body such as Langerhans cells of the skin, Paneth cells in the small intestine, osteoclasts, brain microglia, cells in the female reproductive tract, and at low levels on hematopoietic stem cells (36).

**Figure 2:**
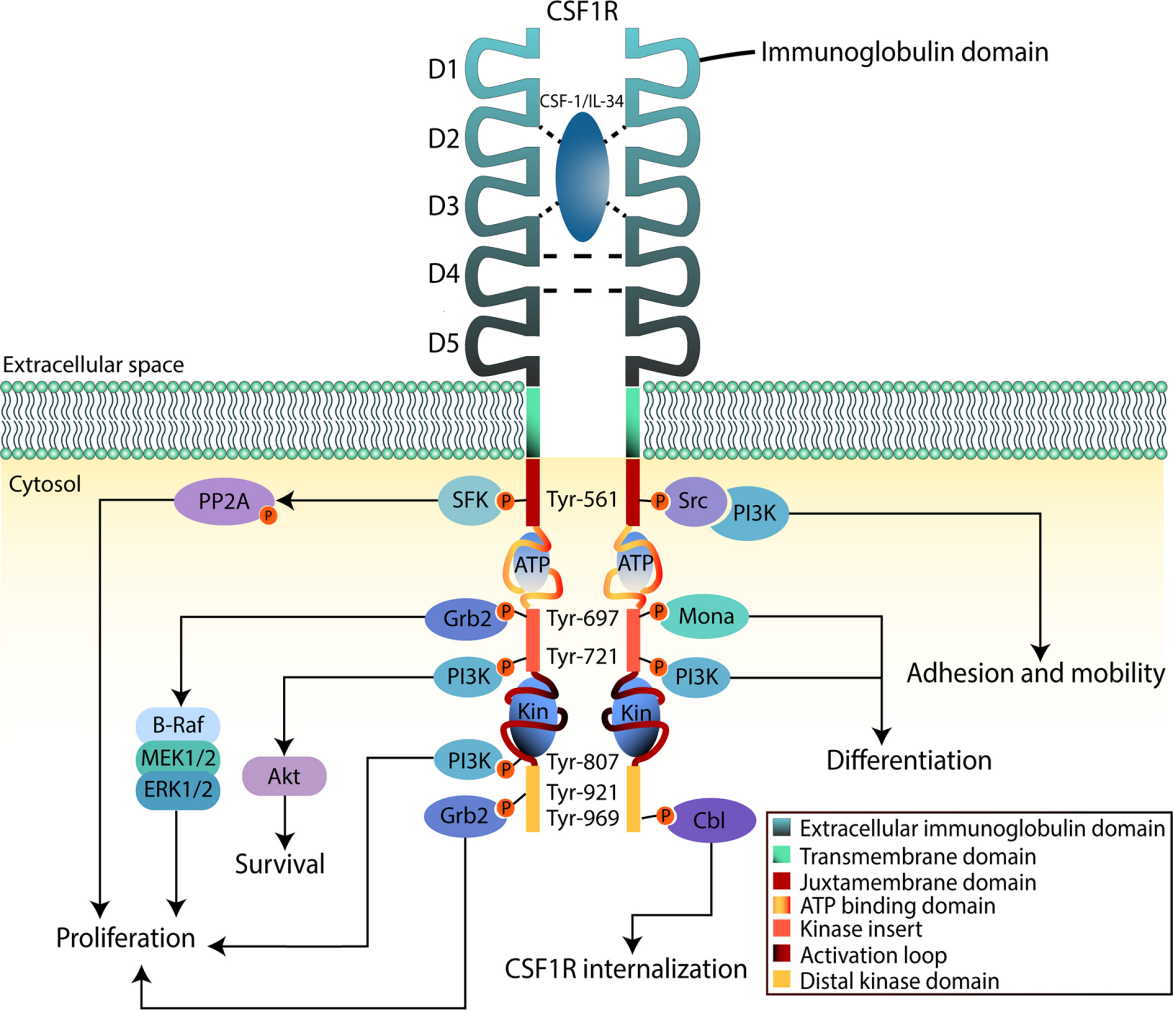
CSF1R downstream signaling in myeloid cells. The binding region of CSF-1 or IL-34 to CSF1R is contained in the second and third domain (D2 and D3) of the extracellular region, D4 mediates homotypic interactions. Upon binding to the ligand, CSF1R dimerizes inducing tyrosine phosphorylation that will lead to activation of downstream signaling pathways. This will promote proliferation, survival, and differentiation of the cell. The intracellular region, apart from the tyrosine residues, contains an ATP-binding domain as well as catalytic domains where substrates bind (Kin). When activation takes place, these domains will fold to phosphorylate signaling mediators.

CSF1R is a cell surface protein with an extracellular glycosylated domain comprising five immunoglobulin (Ig)-like domains (D1-D5), a transmembrane domain and a cytoplasmic kinase domain (**Figure 2**). The intracellular portion of the receptor is composed of eight tyrosine phosphorylation sites situated on the juxtamembrane section, the kinase insert, the major kinase domain, and distal kinase domain (37). In the inactive state, CSF1R presents an autoinhibitory conformation (38). The two activating ligands; CSF1/M-CSF and the more recently identified interleukin 34 (IL34) differ slightly in structure but show undistinguishable downstream signaling pathways according to Boulakirba et al. (39). However, they discovered differences in cytokine/chemokine production when CSF1- or IL34-differentiated monocytes are polarized into different phenotypes. This suggest that macrophages derived from either ligand may behave differently and thus exert different polarization potential. Another study found that CSF1 and IL34 have different spatiotemporal expression but serve complementary roles in regulating the development and maintenance of macrophages (40). Binding of CSF1 or IL34 to CSF1R induces non-covalent dimerization of the receptor chains and transphosphorylation of tyrosine residues (41). The first tyrosine to be phosphorylated is Tyr561 which is necessary for full receptor activation (42). The phosphorylated residues function as docking sites for several different proteins that subsequently activate signaling molecules. Among them are members of the Src family kinases, phospholipase Cγ2, phosphatidylinositol 3-kinase (PI3K), and suppressor of cytokine signalling-1 (SOCS1) (43). Following the different downstream signal transduction pathways, the resulting gene expression mechanisms promote proliferation, differentiation, and survival of the cell (44). Studies analyzing the effects of CSF1R-mutations in macrophages suggest that the PI3K/Akt pathway has a pivotal role in ensuring CSF1-mediated survival of macrophages (40). Macrophage proliferation is primarily associated with the two pathways through PI3K and MEK, but multiple ERK tyrosine kinases may also be involved. Studies have shown that macrophage differentiation is mediated through the PLC-ϒ2 pathway activated by phosphorylation of Tyr-721 and Tyr-807 in CSF1R (40). Knowledge about CSF1R biology and its role in cancer is evolving rapidly, especially regarding the supportive tumor microenvironment.

## CSF1R and the Microenvironment

Bone marrow stromal cells facilitate growth of normal hematopoietic and leukemic cells through the continuous production of growth factors. In AML, malignant cells are thought to polarize the surrounding stroma through a cytokine-regulated mechanism facilitating a stroma-mediated protection of AML. This complex process involves several cytokines, chemokines, growth factors, receptors, and adhesion molecules (45). This can be seen *in vitro* by increased AML cell proliferation and protection from drug-induced apoptosis when these cells are in direct or indirect contact with the human stromal cell line HS-5 (46–48). Thus, targeting the niche and the interaction between leukemic cells and their environment appear promising for AML treatment.

**Figure 3:**
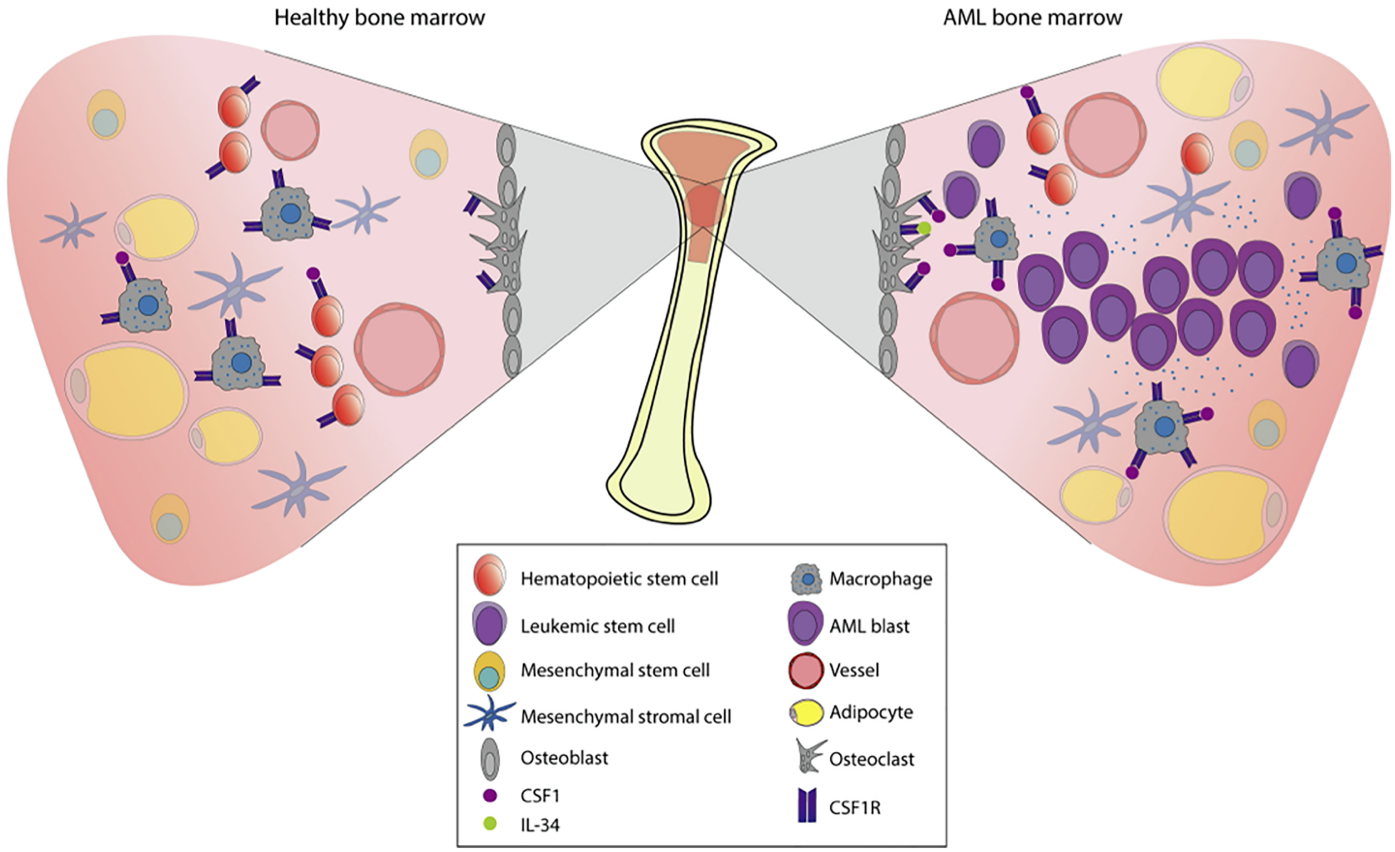
Pathophysiology of AML in the bone marrow. The bone marrow is a tissue organized to protect cell proliferation. Under normal conditions (left), the multipotent hematopoietic stem cells (HSCs) will give rise to the myeloid and the lymphoid lineages. Myeloid lineage transcription factors regulate the expression of CSF1R, already present in macrophage precursors. The stromal environment is composed by osteoblasts, osteoclasts, adipocytes, macrophages, mesenchymal cells, and vessels that will allow this differentiation process to take place. When there is a dysregulation of proliferation and apoptosis in myeloid precursors, hematologic malignancies such as AML may appear. In AML (right), proliferation and survival rely on the bone marrow stromal cells signals. These signals are strongly mediated by the CSF1R which is expressed in certain cell types (shown in the figure), contributing to paracrine communication.

The bone marrow microenvironment (BME) in AML plays an important role by contributing to both leukemic development and therapy resistance. Situated in the BME we find the bone marrow niche; a controlled perivascular and endosteal space where hematopoietic stem cells (HSC) are regulated and maintained by the surrounding stroma (**Figure 3**) (49, 50). The surrounding stroma that affects HSC consists of mesenchymal stromal cells, osteoblasts, endothelial cells, macrophages, and CXCL12-abundant reticular cells in addition to neurons and glial cells (51–56). CSF1R signaling is present in several cellular subpopulations that regulate hematopoiesis and homeostasis. Detectable expression of CSF1R is found mainly on macrophages, osteoclasts, and at low levels on HSC (44). Interestingly, recent studies have revealed CSF1R-expression on leukemic stem cells (LSC) (10), which share phenotypical and functional similarities with HSC. LSC have the capacity to produce a cellular hierarchy of leukemic progenitors as well as remodeling the bone marrow niche, reshaping it into an environment conducive to support leukemic expansion. Thus, the leukemia niche is created where cells are not subject to the same signals as normal HSC and consequently contributes towards malignant progression. More specifically, angiogenesis increases and stromal cells acquire supportive features for the leukemic cell population (57). Failure to eradicate LSC following chemotherapy often contributes to early relapse (58–60).

Another important component that characterizes the AML bone marrow niche includes the interaction between leukemic and endothelial cells. For example, AML cells can intravasate into the vasculature and fuse with endothelial cells thereby creating a favorable vasculature for expansive cell proliferation (61, 62). The level of proangiogenic vascular endothelial growth factor (VEGF) is high in AML patients, and high levels of VEGF and can also lead to increased secretion of granulocyte-macrophage colony stimulating factor (GM-CSF) which is known to stimulate cell growth in AML (63). Fibroblasts also participate in AML development, and studies have found several functional cancer-associated fibroblasts (CAF) in AML patient samples (64). Evidently, all components of the AML bone marrow niche interact with leukemic cells and affect their proliferation, differentiation, adhesion, quiescence, migration, and clonal expansion (45). These interactions between leukemic cells and the BME can determine the fate of leukemic cells following chemotherapy, which and ultimately has implications for residual disease and early relapse (65).

For most intermediate and high-risk AML patients, allogeneic stem cell transplantation is the only curative therapy available (7). Depending on intensity of the transplant conditioning therapy, the bone marrow stroma is more or less damaged by the stem cell transplant process, but donor stem cell engraftment is difficult if the radiation-resistant recipient resident macrophages are eradicated. In an attempt to elucidate this process *in vivo*, mice were inserted with a CSF1R-eGFP construct used as a myeloid reporter gene, where GFP is under the control of the CSF1R promoter. After the stem cell niche was exposed to lethal radiation, subsequent analysis of the niche was performed following lethal irradiation and autologous hematopoietic stem cell transplantation (66). Recipient CD169+ CSF1R-eGFP resident macrophage number in bone marrow aligned with the persistent engraftment of long-term reconstituting HSC within bone marrow, illustrating the complex properties of macrophages in stem cell niches of the bone marrow.

## CSF1R and Tumor-Associated Macrophages

Macrophages are myeloid cells derived from monocytes (67) present in the tumor micromilieu of the bone marrow (68). The major regulators of macrophage proliferation and survival are the growth factors M-CSF/CSF1 and IL34. These play an autocrine/paracrine role in various solid tumors, attracting and differentiating incoming monocytes into tissue resident macrophages in the tumor microenvironment (69–71). Monocytes and macrophages have come to the attention of cancer researchers because of their plasticity and influence on malignant progression as well as their role in cancer-related inflammation (72–76). Increasing evidence demonstrates a correlation between macrophage density within the tumor micromilieu and malignant progression carrying a poor prognosis (77–80).

Macrophages are thought to be polarized by various cytokines towards pro-inflammatory or anti-inflammatory behavior, which means that they exert either influence on tumor development (81). In endometrial and breast cancer, macrophages undergo cancer-specific reprogramming which significantly alters their distribution and function (71). In general, it is thought that a complex bidirectional communication between the macrophages and the tumor ultimately forge the anti-inflammatory, immune regulatory myeloid cells into tumor-associated macrophages (TAM).

To describe macrophage activity, a functional classification of macrophages into M1 and M2 has been proposed, where M1 are pro-inflammatory and tumoricidal, and M2 are anti-inflammatory and immune regulatory. M1 macrophages are differentiated by another growth factor; granulocyte-macrophage colony stimulating factor (GM-CSF, or CSF2) and other pro-inflammatory agents (82). This simple dichotomy excludes the spectra of diverse phenotypes within the tumor microenvironment (82, 83). In response to local microenvironmental cues, TAM display an impressive adaptability that elicit functions supporting tumor growth and resistance to therapy (84). Convincing experimental and clinical evidence has shown that macrophages promote cancer initiation, angiogenesis, migration, and invasion suggesting that specialized subpopulations of macrophages may be important therapeutic targets (85).

Our knowledge of the roles of macrophages and their functions in the development of AML is still limited, although a recent *in vivo* study showed correspondence between macrophage infiltration and overall survival (53). Further, AML cells have also been shown to polarize macrophages and orchestrate the invasion of monocytes into bone marrow of mice, suggesting that AML and surrounding stroma affect monocytic infiltration and transformation into a tumor supportive phenotype. Altogether, the complex interplay between TAM and malignant cells further provides rationale for targeting CSF1R in AML as the receptor is essential for macrophage differentiation and survival.

## CSF1R Mutation and Expression

Recent analyses employing whole-exome and whole-genome sequencing [from the collaborative Beat AML research program and the Cancer Genome Atlas Program’s (TCGA) study on AML] did not detect significant mutational events in CSF1R (10, 19). When we searched for genetic alterations in CSF1R in the accessible databases TCGA (86, 87) and COMSIC (88) we found that 0.9% of all patients (n = 2,034) had CSF1R deletions in one allele. Yet, CSF1R mutations found at codon 301 (L301S) and 969 (Y969F) have earlier been identified in some patients with AML (89). Interestingly, mutations at codon 301 are believed to contribute to constitutive activation of the receptor, while the tyrosine residue at codon 969 have shown to be involved in negative regulatory activity (90). However, the total incidence of mutations in codon 969 was 12.7% and only 1.8% in codon 301. These studies date back to 1990 and included a samples size of 110 patients with myelodysplastic syndromes and AML. Furthermore, studies have revealed that a carboxy-terminal truncation and the two point-mutations (L301S and A374X) in the extracellular D4 domain are crucial for activation of the oncogene (91, 92). In addition, another oncogenic derivative with two translocations and a constitutively active CSF1R fusion protein joined to the carboxy-terminal 399 amino acids is reported in megakaryoblastic AML (FAB classification M7) (93). Conclusively, as recent sequencing analyses did not reveal any significant mutational events in a large sample of AML patients, CSF1R mutations do not appear to be relevant as a target in AML.

Nevertheless, inappropriate expression of CSF1R has been associated with several malignancies, including breast cancer, prostate cancer, ovarian cancer, leukemias, and Hodgkin’s lymphoma (67, 84). More importantly, analysis of CSF1R expression levels in AML patient samples found a correlation between high levels of CSF1R expression and shorter overall survival (94).

Moreover, it has been demonstrated that the runt-related transcription factor 1 (RUNX1), which plays a critical role in the development of AML, is involved in CSF1R transcriptional regulation. RUNX1 plays a key role in the regulation of growth and survival of macrophages by controlling CSF1R gene expression and, in turn, RUNX1 expression is repressed in CSF1-stimulated cells (95). Specifically, RUNX1 regulates expression of RUNX3, CSF1R, and CEBPA genes (96). RUNX1 has also been shown to be a key mediator (directly or indirectly) of tumorigenesis. In BRAF inhibition resistant melanomas, for example, RUNX1 has been shown to autocrinally upregulate expression of CSF1R which possibly contribute to growth and invasion (97). In contrast, RUNX1 loss-of-function mutations in hematopoietic stem progenitor cells (HSPC) reduce rates of apoptosis and increase stress resistance with a consequent selective advantage over normal HSPC (98). Also; a recent investigation showed correlation between inversion of chromosome 16 and CSF1R overexpression in AMl blasts (99).

Further research is needed to determine if specific genetic subsets of AML influence CSF1R expression.

We hypothesize that dysregulation of CSF1R expression through other mutations may benefit from CSF1R inhibitors.

## Preclinical Activity of CSF1R Inhibitors

The complex interaction between leukemic blasts and the surrounding stroma could be exploited therapeutically, as novel treatment in combination with standard treatment regiments. Inhibition of CSF1R has been proposed to be an effective target for blocking monocytes and TAM that infiltrate the tumor stroma and support tumor growth (100). Several companies have produced small molecule inhibitors of CSF1R kinase activity; and most of these have been claimed to be highly specific (44, 100). However, given the high level of conservation of the tyrosine kinase domains of the type III protein tyrosine kinases (CSF1R, Fms-like tyrosine kinase-3, KIT, platelet-derived growth factor receptor), it would be difficult to predict off-target impacts *in vivo*, based on the *in vitro* data.

Edwards and co-workers have recently performed an *ex vivo* functional screen of patient-derived leukemic cells from the “Beat AML consortium,” with the goal of identifying new therapeutic targets (94, 101). Interestingly, small-interfering RNA (siRNA) tyrosine kinome screen identified CSF1R to significantly reduce cell viability in primary AML patient samples. Sensitivity towards CSF1R inhibition (reduced cell viability *ex vivo*) was found in 23% of patient samples. The CSF1R-inhibitor GW-2580 showed high degree of specificity compared with other class III receptor tyrosine kinases and was selected to be CSF1R inhibitor activity in all subsequent experiments. Screening of 315 AML patient samples for sensitivity towards GW-2580 revealed a wide range of responses ranging from highly sensitive to non-sensitive. Nevertheless, GW-2580 significantly induced apoptosis in patient samples but not in samples from healthy donors. Analysis of the patient samples that had undergone inhibitor screening from the “Beat AML” patient cohort revealed a significant association between resistant samples and poor prognostic markers. Cytogenetic abnormalities included complex karyotypes, inversion 3, monosomy 5/deletion 5q, the gene mutations TP53, NRAS, KRAS, and genetic adverse prognostic risk group correlated with increased CSF1R inhibitor sensitivity. The authors concluded that the samples resistant towards CSF1R inhibitors were potentially non-sensitive towards all forms of treatment, which could explain its ineffectiveness.

Examinations of CSF1R expression patterns by flow cytometry and mass cytometry (CyTOF) of samples from AML patients and healthy donors revealed overall expression of CSF1R in AML samples is found on a subpopulation of CD14-expressing monocytes which seem to diminish after exposure to CSF1R inhibitors, while negligible expression of CSF1R was found on a small portion of leukemic blasts (94). In solid tumors, cells expressing CSF1R almost exclusively defines a population of tumor infiltrating macrophages. However, because AML can arise from macrophage-lineage precursor cells, it can be challenging to determine the origin of supportive CSF1R-expressing cells, and to know whether they are infiltrating monocytes/macrophages or tumor derived.

Analyses of CSF1R ligand stimulation suggest that receptor signaling occurs through a ligand-dependent mechanism, and CSF1R inhibitors eliminate CSF1R-expressing supportive cells in AML (94). Furthermore, CSF1R-expressing cells protect AML cells through paracrine cytokine secretion of hepatocyte growth factor (HGF) and CSF1 and that utilizing CSF1R inhibitors may be an effective treatment in a subpopulation of AML patients (94, 101).

It has been hypothesized that using CSF1R inhibitors is most effective in the early stages of the disease (101), which presents an ongoing issue in clinical development as Phase 1 clinical trials usually enroll relapsed/refractory (R/R) AML patients. Increased clinical response in *de novo* disease compared to late-stage cancer is common for many malignancies but studying these patterns requires comprehensive clinical trials. For instance, it took nearly a decade to complete the Phase III registration trial for the first FLT3 targeted treatment in otherwise healthy patients with *de novo* AML (102). Therefore, we expect it will take time to develop CSF1R inhibitors for early stage and first line AML.

## Clinical Development of CSF1R Inhibitors

A wide variety of clinical trials have used different treatment strategies to target CSF1R (**Table 1**). Ongoing studies aim to decipher the safety profile and the clinical activity of CSF1R inhibitors alone and in combination in various malignant diseases. Some of these studies have been previously reviewed by Cannarile et al. (100), describing different approaches for targeting CSF1R in different cancer types. The FDA has recently approved the oral small-molecule CSF1R inhibitor pexidartinib (PLX3397/PLX10801) as monotherapy for the CSF1-driven non-malignant diffuse-type Tenosynovial Giant Cell Tumor (dt-GCT) (103). Pexidartinib also has inhibitory activity against FLT3 and cKIT, two commonly mutated genes in AML. Results of a phase I/II open-label clinical study of pexidartinib in relapsed/refractory (R/R) FLT3-ITD-mutated AML have recently been published (NCT01349049) (104). In this study, 90 patients were treated either in dose escalation or in dose expansion with the aim of assessing safety and tolerability of pexidarnitib, and the maximum tolerated dose (MTD) was not reached. The overall response rate (ORR) was 21%, probably due to patients having received multiple lines of therapy. Although this study focused on treating R/R FLT3-ITD^+^ AML, it also considered the possibility of targeting CSF1R with pexidartinib in patients with wild type FLT3. This study reported tolerability and an antileukemic effect of pexidartinib in highly pretreated R/R AML. Another ongoing study with similar objectives is a Phase I/II trial of pexidartinib in children and young adults with R/R leukemias (AML or ALL) or solid tumors is currently under investigation (105). The results of this trial will help to decipher safety profiles of CSF1R inhibition in the younger population.

**Table 1:** Summary of CSF1R targeted therapies for leukemias in clinical trials (2020).

Compound	Class	Target	Clinical phase	Status	ClinicalTrials.gov identifier	Sponsor
Pexidartinib (PLX3397/PLX10801)	Small molecule	CSF1R, FLT3, cKIT	I/II	Completed [44]	NCT01349049	Daiichi Sankyo, Inc.
			I/II	Ongoing	NCT02390752	National Cancer Institute (NCI)
JNJ-40346527	Small molecule	CSF1R	II	Terminated	NCT03557970	OHSU Knight Cancer Institute
NMS-03592088	Small molecule	CSF1R, FLT3, cKIT	I/II	Ongoing	NCT03922100	Nerviano Medical Sciences
Emactuzumab (RG7155)	mAb	CSF1R	I	Ongoing	NCT02323191	Hoffmann-La Roche

Other CSF1R inhibitors have also been considered for treatment of R/R AML. One example is the selective small molecule CSF1R inhibitor JNJ-40346527, for which preclinical studies have shown interesting immunomodulatory effects in murine models of Crohn’s Disease (106). Although this component’s safety and efficacy have been tested previously in patients with rheumatoid arthritis and advanced Hodgkin’s Lymphoma (107, 108), it has yet to be studied in patients with R/R AML. A phase II open-label clinical trial with the aim of studying efficacy of JNJ-40346527 in patients with R/R AML had to be terminated due to insufficient patient enrolment, unfortunately. The reasons for poor enrolment are unknown, however, it might be increasingly difficult to secure accrual in trials with monotherapy in an aggressive disease like AML (109).

A different approach for treating R/R AML is the ongoing open-label phase I/II, first-in-human clinical trial assessing the clinical activity of the combined FLT3, KIT, and CSF1R inhibitor NMS-03592088 (110). This multi-center non-randomized study aims to assess the safety, tolerability, pharmacokinetics, and pharmacodynamics of NMS-03592088 in patients with R/R AML or chronic myelomonocytic leukemia (CMML) (111). Like pexidartinib, the three targets FLT3, KIT, and CSF1R of NMS-03592088 are connected to the molecular physiopathology of AML, being relevant mediators to target for treatment strategies.

In addition to small-molecule therapeutics, monoclonal antibodies (mAbs) are also being considered as valid options for targeting CSF1R. The most promising example of anti-CSF1R therapy is emactuzumab (RG7155) given as monotherapy for dt-GCT (112). A Phase I clinical trial with emactuzumab in combination with the chemotherapeutic agent paclitaxel in solid tumors reduced TAM at the optimal biological dose (OBD) (113). The efficacy of this monoclonal antibody is yet to be tested in AML, but more clinical data from studies investigating the behavior of emactuzumab (NCT02323191) will give us further knowledge about this agent.

## Discussion

Clinical development of CSF1R inhibitors for AML treatment is in its early development. The activity of some CSF1R inhibitors is modest and more studies are needed to understand the therapeutic potential of these inhibitors. It is important to understand the possible negative side-effects of on-target toxicities like macrophage depletion outside the tumor (114). Similarly, central nervous side effects like fatigue should be considered when investigating CSF1R-targeted therapies for AML (115, 116). A recent study discovered that pexidartinib affects CNS microglia but also has long-term effects in the myeloid and lymphoid compartments of the bone marrow, spleen, and blood (117). The long-term effects on circulating and tissue macrophages have implications for future development of CSF1R inhibition as treatment, because peripheral monocytes repopulate the central nervous system. AML is a highly heterogenic disease with specific therapies, and CSF1R inhibition may represent a more universal approach that targets the stroma. Although single agent sensitivity to CSF1R inhibitor is observed in AML, monotherapy will most likely not be sufficient for efficient AML treatment (94, 101). We suggest a combined approach for targeting the leukemia cells directly as well as the surrounding stroma. For other tumor types, various combinations with CSF1R-mediated TAM depletion are currently under clinical investigation (114). A strategy that may be attractive uses CSF1R inhibitors in combination with a CXCR2 antagonist (118). Kumar and coworkers employed CSF1R inhibition to disrupt chemokine secretion by cancer associated fibroblasts (CAF) abolishing recruitment of pro-tumor granulocytic myeloid-derived suppressor cells (MDSCs). In addition, combining CSF1R inhibitors with a CXCR2 antagonist blocked the infiltration of these cells and showed strong anti-tumor effect (118). CSF1R-targeting agents in combination with checkpoint blockade inhibitors, other targeted therapies, anti-angiogenic therapies, chemotherapy, and adoptive T-cell transfer approaches are all currently undergoing clinical investigations (114). Though several inhibitors are targeting leukemic cells directly by inhibiting FLT3 and TAM by inhibiting CSF1R, we speculate that these inhibitors may be beneficial to use in a carefully designed sequences with immunotherapeutics like immune checkpoint inhibitors.

## Concluding Remarks

The supportive microenvironment in the bone marrow of AML patients significantly contributes to early relapse and death and is a major challenge for successful treatment. The contribution of tumor-associated macrophages (TAM) to malignant progression in AML is substantial, involving bidirectional communication between leukemic cells and TAM. Targeting CSF1R-expressing TAM may be an effective treatment for depleting supportive cells and kill leukemic cells (112, 119). Limited evidence has demonstrated that CSF1R-inhibition has been a beneficial approach for a subset of AML patients (94). At present, the most effective therapy for AML is combinations and sequences of anti-leukemic therapeutics, for example, the recent combination of venetoclax plus hypometylating agents or the sequence of intensive chemotherapy followed by allogeneic stem cell transplantation (120, 121). We suggest a novel approach for eliminating leukemic cells directly and attacking the leukemia-supporting surrounding stroma by inhibiting CSF1R signaling. Future work needs to address the optimal CSF1R targeting combinations and sequences that secure the most clinical benefit for AML patients.

## Author Contributions

All authors listed have made a substantial, direct, and intellectual contribution to the work and approved it for publication.

## Funding

This study was supported by grants from the Norwegian Cancer Society (Grant number: 190175-2017) with Solveig and Ove Lund’s legacy.

## Conflict of Interest

The authors declare that the research was conducted in the absence of any commercial or financial relationships that could be construed as a potential conflict of interest.
